# Central Nervous System Involvement by Small Lymphocytic Lymphoma after a Myxoma-Related Embolic Event

**DOI:** 10.1155/2019/1825491

**Published:** 2019-11-15

**Authors:** Nicolas Gallastegui, Daniel P. Cassidy, Deborah O. Heros, Francisco Vega, Jonathan H. Schatz

**Affiliations:** ^1^Division of Hematology, Department of Medicine, University of Miami Leonard M. Miller School of Medicine/Sylvester Comprehensive Cancer Center, Miami, FL, USA; ^2^Division of Hematopathology, Department of Pathology and Laboratory Medicine, University of Miami Leonard M. Miller School of Medicine/Sylvester Comprehensive Cancer Center, Miami, FL, USA; ^3^Division of Neuro-Oncology, Department of Neurology, University of Miami Leonard M. Miller School of Medicine/Sylvester Comprehensive Cancer Center, Miami, FL, USA

## Abstract

Involvement of the central nervous system by chronic lymphocytic leukemia/small lymphocytic lymphoma is exceedingly rare, and currently no risk factors have been described. We report the case of a patient with concomitant chronic lymphocytic leukemia/small lymphocytic lymphoma and an embolic cerebrovascular accident related to a cardiac myxoma, who developed parenchymal central nervous system involvement of lymphoma on the ischemic bed. The patient was successfully treated with a high-dose fludarabine-based chemotherapy regimen, achieving a sustained remission. We propose that embolic breakage of the blood-brain barrier may be a major risk factor in producing central nervous system involvement. We also propose that a high-dose fludarabine-based chemotherapy regimen may be adequate to achieve a better CNS penetration and improved outcomes.

## 1. Introduction

Chronic lymphocytic leukemia (CLL) and small lymphocytic lymphoma (SLL) are considered the same entity under the spectrum of the B-cell non-Hodgkin lymphomas. Characterized by monoclonal accumulation of CD5/CD19/CD20+ B-cells, the disease is called CLL when presenting with peripheral blood counts exceeding 5.0 × 10^9^/L and SLL when presenting with primarily lymphomatous involvement of spleen or lymph nodes [1].

Infiltration of CLL/SLL cells outside lymphoid tissues, called extramedullary CLL (EM-CLL), is rare. Though a common and potentially devastating complication of aggressive lymphomas like Burkitt lymphoma (BL), fewer than 200 cases of central nervous system (CNS) involvement by CLL/SLL are reported in the literature [2]. Most commonly, it is described in patients with high counts of malignant lymphocytes in peripheral blood [3]. A systematic review by Ratterman et al. found that up to 27% of EM-CLL was in the CNS [4], and Strati et al. reported it carried the worst prognosis of all EM-CLL, with an average survival of 12 months [5].

Cardiac myxomas are the most common type of rare cardiac tumors, constituting 50–85% of lesions in adults and localized to the left atrium in 60–80% of cases [6]. Among myxoma patients with neurologic manifestations, 89% are attributed to ischemic events. Between 30% and 50% of patients with cardiac myxomas manifest peripheral embolization as the initial sign of disease [7]. There is no known association in between myxomas and lymphomas. A prior case series described 12 cases of myxomas associated with non-Hodgkin lymphomas, primarily Epstein–Barr virus-positive nongerminal center diffuse large B-cell lymphomas, and only one was CLL/SLL. Embolic events occurred in two of these cases, and in one, lymphoma cells were found together with the embolic myxoma cells [8].

Here, we present a case of central nervous system involvement by CLL/SLL after an embolic event by a concomitant myxoma.

## 2. Case Description

A 62-year-old male with a history of hypertension and a remote in-situ melanoma treated with resection presented to our institution in May 2016 with acute left upper extremity weakness.

**Figure 1:**
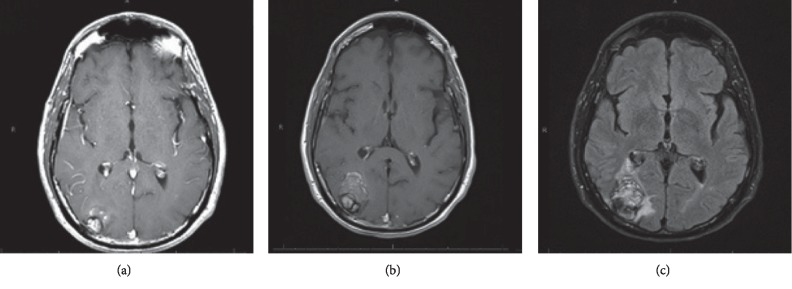
(a) Axial view T1 with Gadolinium at presentation, showing right posterior temporal lesion with a T1 hypointense rim suggestive of atypical stroke. Images of new findings at the previous area of stroke to evaluated new neurologic symptoms: (b) axial view T1 with Gadolinium showing an internal increase in size and (c) axial view FLAIR showing a heterogeneous lesion with hypointense foci within associated surrounding edema and enfacement of posterior horn or right lateral ventricle.

Initial magnetic resonance imaging (MRI) showed multiple mixed signal intensity mass lesions on the right posterior temporal and occipital lobes with areas of hemorrhage and restricted diffusion suggestive of blood products. (Figure 1(a)). Complete blood count (CBC) showed normal white blood cell count at 8.2 × 10^3^/*μ*L (range: 4–10.5 × 10^3^/*μ*L) with normal differential and normal absolute lymphocyte count, as well as normal hemoglobin at 12.9 g/dl (range: 13–16 g/dl) and platelet count at 249 × 10^3^/*μ*L (range: 140–400 × 10^3^/*μ*L).

Initial suspicion was for embolic cerebrovascular accident (CVA) or less likely CNS metastasis. Positron emission tomography-computed tomography (PET-CT) performed to assess for underlying neoplasm, showing a low-grade fluorodeoxyglucose (FDG) avid uptake (standardized uptake value 2.8) over left atrial mass and subcentimeter non-FDG avid axillary and mediastinal lymphadenopathy without additional suspicious lymphadenopathy in the abdomen or pelvis. Computed tomography (CT) scan of the chest, abdomen, and pelvis with intravenous contrast showed bilateral axillary nodules up to 1.9 cm and scattered nonsuspicious subcentimeter lymph nodes in the abdomen and pelvis.

Echocardiogram showed a 2.5 cm globular mass on the left atrium highly suspicious for myxoma. Cardiac MRI corroborated a polypoid mobile mass from the intra-atrial septum, measuring 2.5 × 4.0 × 2.8 cm. The patient was managed conservatively as neurologic symptoms improved without intervention. An elective cardiac myxoma resection was recommended to remove the presumed source of emboli. One month later, the patient underwent cardiac mass resection, with pathology confirming cardiac myxoma.

Two months after the resection the patient was evaluated for progressive growth of tender subcutaneous nodules of 1-2 mm on the distal left fifth digit and distal right fourth digit. He underwent resection on November 2016. Pathology review reported myxoma from cardiac source. At this time, CBC showed normal white blood cell count at 4 × 10^3^/*μ*L with normal differential and normal absolute lymphocyte count, as well as normal hemoglobin at 13.4 g/dl and platelet count at 158 × 10^3^/*μ*L. The new myxoma lesions were presumed metastases from the already resected primary tumor. No additional workup was performed.

The patient was stable and followed clinically until May of 2017 when he developed gradual onset of intermittent neurologic symptoms characterized by dysmetria, left upper extremity paresis, apraxia, mild amnesia, and prosopagnosia. The patient underwent evaluation by his primary care doctor who recommended a brain MRI, showing a right parieto-occipital mass (Figure 1(b)). On physical exam, he had palpable bilateral inguinal lymphadenopathy up to 2 cm. The patient reported noticing these nodes up to 2 months previously and reported intermittent cervical and inguinal lymphadenopathy for 2 years. CBC showed normal white blood cell count at 5.3 × 10^3^/*μ*L with normal differential, as well as normal hemoglobin at 13.3 g/dl and platelet count at 170 × 10^3^/*μ*L.

The patient underwent craniotomy with resection in July 2017. The patient's neurologic symptoms improved progressively over a period of two weeks after surgery.

**Figure 2:**
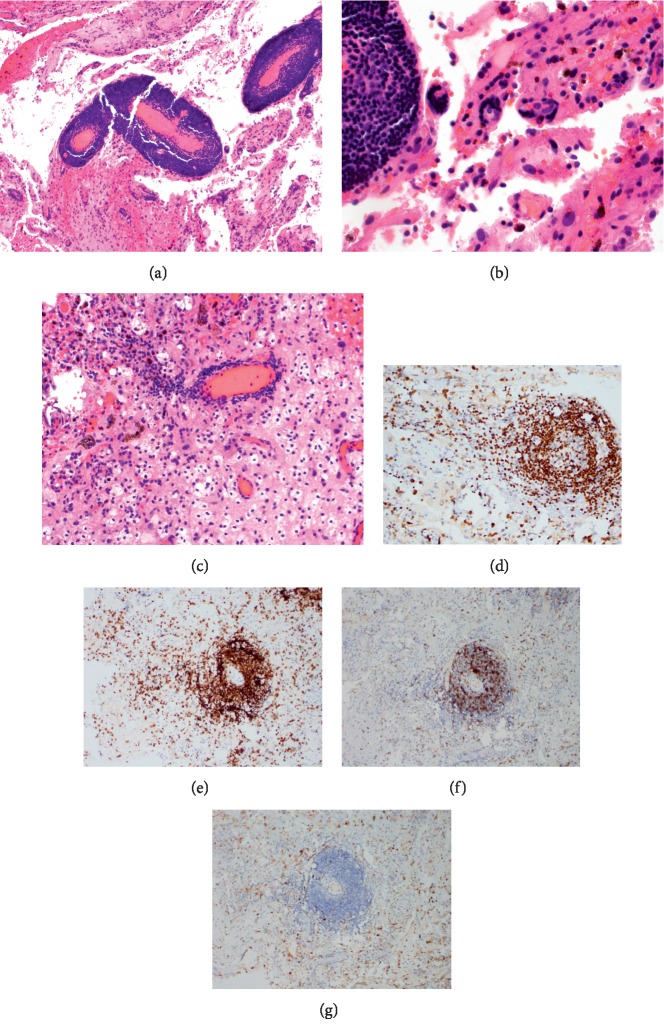
CNS biopsy: (a) lymphomatous infiltrate is predominantly perivascular that pushes into the surrounding brain parenchyma, which is hemorrhagic with numerous hemosiderin-laden macrophages. Intermediate and high-power photomicrographs of the perivascular lymphoid infiltrate: (b) the infiltrate is composed predominantly of lymphoid cells, which are small with mature nuclear chromatin and regular, rounded nuclear borders. (c) More invasive, rather than pushing, infiltrate involving the brain parenchyma. Immunohistochemistry: (d) PAX-5, (e) coexpression of CD5, (f) partial coexpression of CD23, and (g) cyclin-D1 is negative in the neoplastic lymphoid cells.

Unexpectedly, the biopsy showed SLL on an extensively hemorrhagic background. Lymphoma cells were small with a largely perivascular distribution but were also seen in small aggregates throughout the biopsy. No cardiac myxoma was present in the resected tissue. By immunohistochemistry, the lymphoma cells stained positive for CD5, PAX-5, and CD23 and negative for CD20, CD3, BCL6, and cyclin-D1. (Figure 2).

A bone marrow biopsy in October 2017 showed CLL/SLL characterized by small cell atypical infiltrates accounting for 90% of bone marrow cellularity and reduced trilineage hematopoiesis. By immunohistochemistry, atypical cells were positive for CD5, PAX-5, CD20 (dim), and CD23 and negative for CD3, BCL6, and cyclin-D1. Flow cytometry showed a monoclonal B-cell population positive for CD19, CD20 (dim), CD5, DC23, CD38, and dim kappa light chain restriction. Ki67 was positive in 3%. Karyotype was normal, but deletion 13q14 was detected by fluorescent in-situ hybridization, and a molecular profile was positive with IgVH mutation >2% and negative for TP53 mutation. Cerebrospinal fluid was negative for malignancy by cytology and flow cytometry.

Peripheral flow cytometry showed a monoclonal kappa light chain restricted (dim) B-cell population positive for CD19, CD20 (dim), CD5, CD38, and CD23 and negative for CD10. In sum, the picture was consistent with good-risk CLL/SLL.

Rereview of the pathology slides from the previously resected cardiac myxoma and its distal embolic complications did not reveal the presence of lymphoma cells.

The case was reviewed by the expert panel at our institution. Because monoclonal B-lymphocytes in the peripheral blood were less than 5.0 × 10^9^/L, the disease was characterized as SLL Lugano stage IV with extralymphatic extension to the CNS. Although the patient did not meet standard criteria for treatment, the expert panel felt a local recurrence in the CNS could be catastrophic and recommended treatment with systemic therapy that penetrates CNS. The patient underwent treatment FCR chemoimmunotherapy (rituximab, cyclophosphamide, and fludarabine) every 28 days. We increased the dose of fludarabine to 30 mg/Kg from the standard 25 mg/Kg to increase penetration to the CNS. The patient completed three cycles by January of 2018 with complete resolution of palpable inguinal lymphadenopathy after the first cycle. The original plan was to complete four cycles, but the patient declined the fourth treatment.

Over time, the patient developed a focal temporooccipital epilepsy and residual left homonymous hemianopia and presumed secondary to previous resection and remains under management by neurology. No paraneoplastic workup for seizures has been performed. Multiple CSF studies have been negative for malignancy by flow cytometry and cytology. Serial brain MRIs for surveillance have shown no evidence of recurrent tumors out to 12 months after completion of chemotherapy.

## 3. Discussion

Secondary involvement of the central nervous system (CNS) by non-Hodgkin Lymphoma (NHL) is a well-described phenomenon, estimated to happen in up to 5% of all subtypes [9]. The main risk factor overall is histology, with frequency as low as 2.8–5.3% for low-grade lymphomas [10–12] and as high as 30–50% for BL [13]. Other factors such as bone marrow involvement, retroperitoneal extension, and particular extranodal sites (breast, nasal/paranasal, testicular, gynecological, kidney, and adrenals) carry known increased CNS involvement risks for aggressive lymphomas [14]. CNS involvement by NHL is most commonly leptomeningeal, ∼64% in the relapse setting, but up to 28% of cases are purely parenchymal (with some changes in this pattern emerging in the postrituximab area) [15].

In a large database analysis of low-grade lymphomas involving the CNS, CLL/SLL was found to be the third largest group with 8%, in comparison with follicular lymphoma with 38% [12]. The latter, despite being the most common leukemia diagnosed in the US with an annual incidence of 5 cases per 100,000 [16]. CNS involvement by CLL/SLL is, therefore, a rare phenomenon, and no specific risk factor has been established. The mean age of presentation is 64.5 and is more common in males than in females [2].

Overall, CNS infiltration by CLL/SLL has been estimated at 0.4%–2% [3, 5], but these numbers are discrepant with autopsy series in which involvement has ranged from 7–71% [17], suggesting most cases are not clinically significant.

Risk factors for CNS involvement by CLL/SLL are minimally studied [18]. Surprisingly, Moazzam et al. found involvement of CNS was more frequent in patients with a RAI staging of 0 compared with more advanced stages [17], but an autopsy series suggested increased frequency at later stagers and with increased number of organs involved [19]. Disease volume is therefore not strongly linked to CNS involvement, and additional unknown tumor- or host-specific factors may be at play.

Symptoms of CNS involvement by CLL/SLL are nonspecific, with neurocognitive changes being the most common finding to prompt a workup [5]. Cranial nerve-related symptoms result commonly from CNS involvement, part of an overall constellation of headaches, cerebellar symptoms, altered mental status, and cognitive decline [2]. Workup for CNS involvement during initial evaluation of newly diagnosed CLL/SLL is not routine, as only 4% of patients prompted a workup to clarify neurologic symptoms in a large retrospective cohort analysis at a single institution, and from this group, only 10% were found to be positive [5].

Mechanisms by which CLL/SLL cells infiltrate the CNS remains undescribed clinically, though investigators have proposed transmigration through perforating cerebral vessels to the subarachnoid space and/or direct extension from seeded meninges or via perineural sheaths of cranial nerve roots [19]. The frequency of cranial nerve-related symptoms suggests perineural invasion of these nerves is important in many cases.

In this case, the patient had clinical presentation and MRI findings strongly consistent with embolic stroke, and workup revealed a cardiac myxoma, which was later known to have metastasized embolically to distal vascular beds in his fingertips. This history is, therefore, strongly consistent with embolic myxoma leading to his initial CVA and later CLL invastion to the same site, though we cannot definitively exclude the presence of CNS CLL upon initial presentation since CNS resection was not performed at that time.

To our knowledge, our report represents that first case of CNS involvement of CLL/SLL in the setting of a previous embolic event and the second report published of CLL/SLL co-occurring with a cardiac myxoma [20]. The parenchymal location restricted to the embolic bed is highly suggestive that the prior myxoma embolism was a key predisposing factor in this case, contrasting with clinical and autopsy reports that describe the leptomeningeal involvement as most frequent [21], though parenchymal disease is certainly also described [5].

Our report is also unusual in that the patient's presentation required emergent surgical intervention for an acute mass effect. Limb paralysis/paresis was found in only 12% of cases previously, with CNS involvement more commonly an asymptomatic incidental finding and generally arising 30 months or longer after initial CLL diagnosis [2].

No evidence of myxoma cells could be identified in the area of the brain embolism in this case, and no lymphoma cells were identified associated with the primary myxoma or its metasteses, supporting the idea that the prior emboli were enabling factors for lymphoma-cell entry to the CNS. Overall, we believe the interruption of the blood brain barrier was the major precipitating event, allowing the hematogenous spread of lymphoma cells.

We chose FCR treatment because high-dose fludarabine is known to penetrate CNS, based on neurotoxicity studies [22]. We decided to proceed with the standard FCR combination days 1 to 3, although with an increase of fludarabine from 25 mg/m^2^ to 30 mg/m^2^ to increase CNS penetration. Knop et al. [22] reported use of single-agent fludarabine 25 mg/m^2^ (five days per cycle) in two patients with leptomeningeal CLL involvement, achieving remission for 11 months after 6 cycles and 25 months after 5 cycles, respectively. Elliot et al. [23] reported a six-month remission in a patient with extensive leptomeningeal CNS involvement using single-agent fludarabine 30 mg/m^2^ from days one to five for six cycles. Wanquet et al. [24] also showed durable responses on CLL/SLL involving the CNS with fludarabine-based regimens. We also considered ibrutinib, which has adequate levels of brain distribution and has successfully been used as a single agent in the management of other types of lymphoma in the CNS [25] and specifically showed complete response as a second line therapy in a small retrospective cohort of CLL/SLL involving the CNS [24]. Our rationale was to save ibrutinib in case of later recurrence.

The patient here has sustained CNS remission so far as indicated by sequential imaging and clinical monitoring, providing anecdotal further support for fludarabine-containing chemotherapy for CLL cases in which CNS-directed therapy is indicated. Because CLL/SLL and CNS are most common in the elderly, we also suggest that in patients with CLL/SLL, CNS embolisms or other cerebrovascular accidents (CVAs) damaging the blood-brain barrier are potential risk factors for CNS invasion. CNS involvement by CLL/SLL should, therefore, be on the differential diagnosis for patients with CVA history and new neurologic signs or symptoms.
